# The value of knowledge accumulation on climate sensitivity uncertainty: comparison between perfect information, single stage and act then learn decisions

**DOI:** 10.1007/s11625-018-0528-7

**Published:** 2018-01-24

**Authors:** Shunsuke Mori, Hideo Shiogama

**Affiliations:** 1 Tokyo University of Science, Yamasaki 2641, Noda-shi, Chiba 278-8510 Japan; 2 National Institute for Environmental Studies, 16-2 Onogawa, Tsukuba-City, Ibaraki 305-8506 Japan

**Keywords:** ATL multi-stage decision-making, Observational constraint, Climate sensitivity uncertainty, Value of learning, Integrated assessment model

## Abstract

In COP21 followed by the Paris Agreement, the world is now seriously planning actions to mitigate greenhouse gas emissions toward a “below 2 °C above preindustrial levels” future. Currently, we are still far from identifying the emission pathways to achieve this target because of the various uncertainties in both climate science and the human behavior. As a part of the ICA-RUS project, conducted by Dr. Seita Emori of the National Institute for Environmental Studies we have studied how these uncertainties are eliminated by the accumulation of scientific knowledge and the decision-making processes. We consider the following three questions: first, when and how will the uncertainty range on the global temperature rise be eliminated, second which global emission pathway should be chosen before we get the perfect information, and third how much expenditure is justified in reducing the climate uncertainties. The first question has been investigated by one of the authors. Shiogama et al. (Sci Rep 6:18903, 2016) developed the Allen–Stott–Kettleborough (ASK) method further to estimate how quickly and in what way the uncertainties in future global mean temperature changes can decline when the current observation network of surface air temperature is maintained. Fourteen global climate model results in CMIP5 (CMIP http://cmip-pcmdi.llnl.gov/, 2017) are used as virtual observations of surface air temperature. The purpose of this study is to answer the remaining two questions. Based on the ASK research outcomes, we apply the multi stage decision-making known as Act Then Learn (ATL) process to an integrated assessment model MARIA which includes energy technologies, economic activities, land use changes and a simple climate model block. We reveal how accumulating observations helps to mitigate economic losses by expanding the existing ATL method to deal with the uncertainty eliminating process by ASK. The primary findings are as follows. First, the value of information largely increases as the climate target policy is more stringent. Second, even if the uncertainties in the equilibrium climate sensitivity are not fully resolved, scientific knowledge is still valuable. In other words, the expenditure for scientific researches is rationalized when we really concern the global climate changes.

## Introduction: the ICA-RUS project and three questions concerning climate uncertainty

The 2015 United Nations Climate Change Conference (also known as COP21), followed by the Paris Agreement stated that “Emphasizing with serious concern the urgent need to address the significant gap between the aggregate effect of Parties’ mitigation pledges in terms of global annual emissions of greenhouse gases by 2020 and aggregate emission pathways consistent with holding the increase in the global average temperature to well below 2 °C above preindustrial levels and pursuing efforts to limit the temperature increase to 1.5 °C above preindustrial levels” (UNFCC 2015). However, current scientific knowledge is far from identifying the emission pathways to achieve this target. Uncertainties in the projections of global mean temperature change by global climate models (GCMs) become uncertainties in mitigation pathways holding the 1.5 and 2.0 °C targets. Under such uncertainties in the climate changes, the following three questions should be raised: first, when and how will the uncertainty range on the global temperature rise be eliminated, which global emission pathway should be chosen in the absence of perfect information, and how much expenditure is justified in reducing the climate uncertainties.

Most existing mitigation studies use range of uncertainty of climate projections (or climate parameters such as climate sensitivity) obtained from ensembles of GCMs. However, the uncertainty range of the future global mean temperature changes (Δ*T*) is expected to decline in the future thanks to new observations, greater warming signals, and further progress in understanding the climate system. Previous studies have investigated how the effects of possible future learning about climate change might affect mitigation analyses (Manne and Richels 1992; Yohe et al. 2004; Webster et al. 2008; Mori et al. 2013; Neubersch et al. 2014). Most of the studies of this type have used idealized assumptions regarding learning speed. For instance, Yohe et al. (2004) assumed that we would have perfect knowledge about climate sensitivity in the 2030. Some studies have used simulations of Δ*T* from simple climate models as pseudopast and future observations to investigate possible future learning about Δ*T*. Their results are sensitive to the assumption of internal climate variability which cannot be simulated using simple models and prior distributions of climate parameters including climate sensitivity (Webster et al. 2008; Olson et al. 2013; Urban et al. 2014).

Uncertainties in climate change issues are not only the future global mean temperature changes (Δ*T*). For example, it is uncertain how climate change might impact the natural biosphere, agricultural production and human society, which are critical issues in policy making. The development and implementation of energy technologies as well as their societal acceptance are key factors, especially relation to carbon capture and storage (CCS) and geo-engineering options. In this sense, climate change issues should be discussed from the perspective of risk management involving multiple research fields. The Japanese Ministry of the Environment established an inter-disciplinary research project, entitled “Integrated Climate Assessment-Risks, Uncertainties, and Society (ICA-RUS)” conducted by Dr. Seita Emori of the National Institute for Environmental Studies (NIES) for the period of 2012–2016. The purpose of the ICA-RUS project is to provide a basis for the social deliberation on long-term climate goals by exploring advantages and disadvantages involving different targets from a risk management perspective. ICA-RUS attempts to integrate insights from areas of climate risk assessment, energy economics modeling, the energy–water–food–ecosystem nexus, and science and technology studies. The objective of ICA-RUS is first to set a mitigation target (including 1.5 and 2.0 °C), and then assessing the consequences and their ranges. Climatic, mitigative, and socioeconomic uncertainties are then considered. ICA-RUS involves climate science, engineering, economics, and sociology to integrate the climate change impacts, mitigation options, and societal acceptance of stakeholders as a risk management approach. Further details of ICA-RUS are described by Emori et.al. (2017) and ICA-RUS-Reports (NIES 2013, 2014, 2015).

As part of the ICA-RUS project, we focus on how the uncertainty in climate sensitivity should be resolved. Shiogama et al. (2016) developed a novel method and first provided plausible estimate of future learning about Δ*T* to answer the first question in the opening paragraph of this section.

This study aims to answer remaining two questions: (1) which global emission pathway should be chosen in the absence of perfect information based on the estimate of future learning and (2) how much expenditure is justified in reducing the climate uncertainties.

## Method: future observational constraints and the ATL decision-making process

### Observational constraints on future climate change

When considering future climate changes, we require standardized plural scenarios on the climate control target to compare the model results. The climate research community has, therefore, developed four possible greenhouse gas concentration pathways under different climate control policies. These are known as representative concentration pathways (RCPs) (Collins et al. 2013; Vuuren et al. 2011) where the radiative forcing in 2100 is constrained to 8.5 W/m^2^ (RCP8.5), 6.0 W/m^2^ (RCP6.0), 4.5 W/m^2^ (RCP4.5) and 2.6 W/m^2^ (RCP2.6). However, future climate change projections contain intrinsic uncertainties. A method was proposed to constrain the uncertainty in Δ*T* by evaluating GCMs’ climate simulations and comparing them with the historical observations of surface air temperature (Allen et al. 2000; Stott and Kettleborough 2002). This is named Allen–Stott–Kettleborough (ASK) method. Its basic idea is simple: if GCM overestimates the observed magnitude of historical climate change, it will overestimate future climate change by a proportional amount, and vice versa. The future projections of Δ*T* are scaled up or down by this proportional amount, and the uncertainty ranges due to the internal climate variability are estimated.

Shiogama et al. (2016) considered simulations of the Coupled Model Intercomparison Project Phase 5 (CMIP5) (CMIP 2017; Collins et al. 2013) as pseudopast and future observations and applied the ASK method to estimate how fast and in what way the Δ*T* uncertainties can decline when the current observation network of surface air temperature is maintained. Shiogama et al. (2016) investigated the rate of decline of the Δ*T* uncertainty until the end of this century for each of RCPs, and found that more than 60% of the Δ*T* uncertainty in the 2090s (2090–2099) can be resolved by the observation until 2049.

**Fig. 1:**
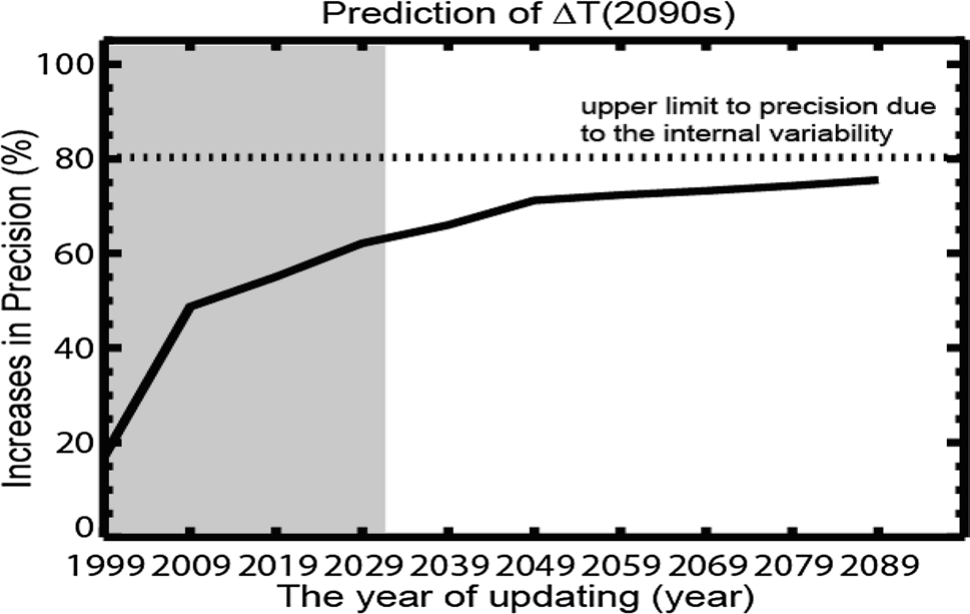
The future fractional decline (% precision) of the Δ*T* uncertainty range estimated in the 2090s relative to the uncertainty range in 14 GCMs. The horizontal axis indicates the update year of the observed data. Within the gray area (up to 2029), the observation data are insufficient for predicting Δ*T* satisfactorily in the 2090s (i.e., the ASK method may fail to accurately constrain Δ*T* in the 2090s). From 2039 onward, we can accurately reduce the Δ*T* uncertainty in the 2090s. The dotted line is the upper limit of precision determined by the internal-climate variability. Beyond this limit, the precision of the ASK method can be improved no further

We apply the same method as Shiogama et al. (2016) to estimate the future decline of the Δ*T* uncertainty in the 2090s using all the four RCPs, whereas Shiogama et al. (2016) analyzed each of four RCPs, respectively. Figure 1 shows the decline of Δ*T* uncertainty by the 2090s thanks to the update of observations. The uncertainty range of Δ*T* rapidly decreases as the more observation data accumulate. We can accurately reduce more than 60% of the Δ*T* uncertainty in the 2090s by 2039 and about 80% by 2089. The reduction rate of the Δ*T* uncertainty is improved because of the increase in the analyzed GCM data size. Shiogama et al. (2016) concluded that 60% of the Δ*T* uncertainty will be reduced by 2049, but that occurs by 2039 in this study. Although Shiogama et al. (2016) proposed a method for how observations reduce the future temperature rise uncertainty, they did not touch upon the mitigation strategies and actions. The present paper investigates the pathways of mitigation option implementations and evaluates the value of observation applying the expanded ATL method to the integrated assessment model MARIA.

### ATL decision-making

**Fig. 2:**
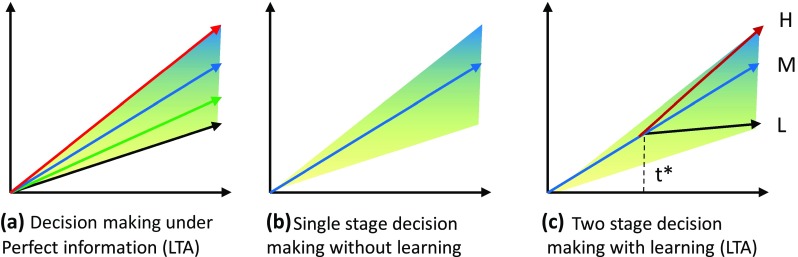
Different decision-making frames under uncertainties. Shaded regions indicate the uncertain range of a certain parameter that spreads from low values to high values at the initial time. The shades are graduated from blue (*H* high-value case), via medium (*M* medium-value case), to yellow (*L* low-value case)

We applied the above uncertainty-decreasing process to multi-stage decision-making. This is known as the Act Then Learn (ATL) procedure, and was first applied to the GLOBAL 2100 model (Manne and Richels 1992). Figure 2 shows the decision-making frames under uncertainties of (a) perfect ex ante information [Learn Then Act (LTA)] decision-making, (b) single-stage decision-making without learning and (c) multi-stage decision-making the ATL learning process. If the future uncertainty is completely resolved prior to the decision-making at the initial time, then the decision maker can select the optimal strategy corresponding to the foreseeable future [case (a)]. On the contrary, if no opportunity to revise the plan arises after the decision-making, the policy maker must select the initial action that maximizes a given objective function such as the expected discounted utility [case (b)]. If the policy maker can change the action based on the learning procedure at an intermediate time t*, as in case (c), the opportunity for change will be considered when deciding the action before t*.

We formulate the model description to address the above decision-making procedure. Let **x**(*t*) and **a** be the control variable at time *t* and parameter with uncertainty, respectively. We define the uncertainty as a set of discrete scenarios *S* = {*s*}, where each scenario s has a probability *P*(*s*) at *t* = 0 and **a**(*s*) represents the parameter **a** in a scenario s. The objective function to be maximized is represented by *f*(**x**(*t*) | **a**(*s*)). If the perfect ex ante information is available at *t* = 0, then we need only to determine the optimal decision under the certain scenario s^*^. This is represented by the case (a) of Fig. 2. The optimal behavior x^*^(*t* | s^*^) is given by the optimal solution of

1 $$\mathop {\hbox{max} }\limits_{{{\mathbf{x}}(t)}} .\quad f({\mathbf{x}}(t|s*)|{\mathbf{a}}(s*)).$$

By contrast, if we know only the future occurrence probability of the scenario s, i.e., *P*(*s*), and the decision can be given only once at *t* = 0, we must explore the optimal pathway **x**(*t*) considering all future possible scenarios. In this case, control variables **x**(*t*) should be identical across the future scenarios as shown in the case (b) of Fig. 2. When we maximize the expected objective function, the optimal pathway x^***^(*t*) is obtained by solving

2 $$\mathop {\hbox{max} }\limits_{{{\mathbf{x}}(t)}} .\quad \sum\nolimits_{{\,s}} {P(s)f({\mathbf{x}}(t)|{\mathbf{a}}(s))} .$$

Although other decision criteria (e.g., minimax regret strategy and maxmax strategy) are also applicable (Mori et al. 2013), we focus herein on the maximum expected value similar to Manne and Richels (1992).

In case (c) in which two-stage decision-making is available, the control variables **x**(*t*) should be identical before t* but can diverge after t*. If the uncertainty set *S* is partitioned into *K* subsets after t*, the optimal solution can be formulated as follows:

3 $$\begin{aligned} & \mathop {\hbox{max} .}\limits_{{{\mathbf{x}}(t)}} \;\sum\nolimits_{{{\kern 1pt} s}} {P(s)f({\mathbf{x}}(t|s)|{\mathbf{a}}(s))} , \\ & {\text{subject}}\;{\text{to}}\;{\mathbf{x}}(t|s)={{\mathbf{x}}_1}(t)\quad {\text{for}}\quad t \leqslant {t^*}, \\ & \quad \quad \quad \quad {\mathbf{x}}(t|s)={{\mathbf{x}}_2}(t|{\mkern 1mu} {R_k}(s))\quad {\text{for}}\quad t>{t^*}\quad k=1,2, \ldots K. \\ \end{aligned}$$

where *R*_*k*_(*s*) represents the *k*th subinterval of *S*. Let x^**^(*t*|*s*) denote the optimum solution of Eq. (3).

For example, future population growth rates up to 2100 are categorized into *S* = {very low, low, middle, high, very high}, but information on the actual rate is lacking. In 2050, the future population growth might be recognized as *R* = {{very low, low}, {middle}, {high, very high}}. If the future population post-2050 is narrowed to {very low, low}, the decision-making can exclude the other possibilities.

We define the value of “information” or “scientific knowledge” by comparing the simulated GDPs in the above three cases in this study according to Manne and Richels (1992).

For instance, the difference between the expected optimal GDP under ex ante perfect information and the expected GDP of the single-stage decision-making gives the “value of perfect information”. Let *Y*(***x***(*t*) | **a**(*s*)) be the GDP of period *t* under the future scenario *s*. The expected value of perfect information at period *t*, namely VPI(*t*), and the discounted summation of the differences, namely TVPI, are defined as

4 $${\text{VPI}}(t)=\sum\nolimits_{{\,s*}} {P({s^*})Y({{\mathbf{x}}^*}(t|{s^*})|{\mathbf{a}}({s^*}))} - \sum\nolimits_{{\,s}} {P(s)Y({{\mathbf{x}}^{***}}(t)|{\mathbf{a}}(s))} .$$

5 $${\text{TVPI}}=\sum\nolimits_{t} {{{(1 - d)}^t}\left[ {\sum\nolimits_{{\,s*}} {P({s^*})Y({{\mathbf{x}}^*}(t|{s^*})|{\mathbf{a}}({s^*}))} - \sum\nolimits_{{\,s}} {P(s)Y({{\mathbf{x}}^{***}}(t)|{\mathbf{a}}(s))} } \right]} .$$

where *d* denotes the discount rate.

The ratio of VPI(*t*) to GDP, namely VPIR(*t*), and ratio of TVPI to the discounted summation of GDP in case (c), namely TVPIR are, respectively, defined as

6 $${\text{VPIR}}(t)=\frac{{{\text{VPI}}(t)}}{{\sum\nolimits_{{\,s}} {P(s)Y({{\mathbf{x}}^{***}}(t)|{\mathbf{a}}(s))} }}$$

7 $${\text{TVPIR}}=\frac{{{\text{TVP}}I}}{{\sum\nolimits_{t} {{{(1 - d)}^t}\sum\nolimits_{{\,s}} {P(s)Y({{\mathbf{x}}^{***}}(t)|{\mathbf{a}}(s))} } }}$$

which represent the ratio of that economic gain of perfect information to the economic output without learning under the initial information.

Similarly, the difference between the GDPs calculated by the optimal solutions of Eq. (3) in case (c) and Eq. (2) gives the accumulated knowledge value of the learning process, namely VLP(t):

8 $${\text{VLP}}(t)=\sum\nolimits_{{\,s}} {P(s)Y({{\mathbf{x}}^{**}}(t|s)|{\mathbf{a}}(s))} - \sum\nolimits_{{\,s}} {P(s)Y({{\mathbf{x}}^{***}}(t)|{\mathbf{a}}(s))} .$$

The discount summation of VLP(*t*), namely TVLP, represents the total value of learning.

9 $${\text{TVLP}}=\sum\nolimits_{t} {{{(1 - d)}^t}\left[ {\sum\nolimits_{{\,s}} {P(s)Y({{\mathbf{x}}^{**}}(t|s)|{\mathbf{a}}(s))} - \sum\nolimits_{{\,s}} {P(s)Y({{\mathbf{x}}^{***}}(t)|{\mathbf{a}}(s))} } \right]} .$$

Similar to Eqs. (6) and (7), we can define the value of learning as follows:

10 $${\text{VLPR}}(t)=\frac{{{\text{VLP}}(t)}}{{\sum\nolimits_{{\,s}} {P(s)Y({{\mathbf{x}}^{***}}(t)\,|\,{\mathbf{a}}(s))} }}$$

11 $${\text{TVLPR}}=\frac{{{\text{TVLP}}}}{{\sum\nolimits_{t} {{{(1 - d)}^t}\sum\nolimits_{{\,s}} {P(s)Y({{\mathbf{x}}^{***}}(t)|{\mathbf{a}}(s))} } }}$$

In previous applications of this approach to integrated assessment models (Manne and Richels 1992; Mori et al. 2013), the uncertainty was eliminated by hypothetical processes. Herein, we investigate the impact of knowledge accumulation on the policies adopted for energy technology. We apply the learning process of Fig. 1 to an integrated assessment scheme named the Multiregional Approach for Resource and Industry Allocation (MARIA) (Mori et al. 2013). We also analyze the economic benefits of the knowledge accumulation.

It should be noted that the concept of “value of information” or “value of scientific knowledge” is extremely broad. It extends from conventional cost-and-benefit analysis approach employed herein to technological and societal innovation as yet unknown. For instance, few people in the previous century could have imagined today’s progress in the information technology or artificial intelligence. Such new knowledge or a big innovation would substantially alter the policies on climate change. However, we cannot evaluate these values nor can we rely on such advance to provide today’s decision. However, there are some possible options with expected large potential and high barriers (e.g., nuclear fusion reactor, space solar power systems (SSPS), and geo-engineering options). The method described herein might be applicable to evaluating these “uncertain” options.

## Expansion of ATL method and application to MARIA model

### Outline of MARIA model

The above method was applied to the MARIA integrated assessment model (Mori 2000; Mori and Saito 2004). This was originally developed as an inter-temporal optimization model that integrates top-down macroeconomic activity with bottom-up technology flows, similar to the GLOBAL 2100 model (Manne and Richels 1992). It also includes a land-use change block in a food demand-and-supply scenario (Mori and Takahashi 1999) and a simple climate model similar to DICE-2013R (Nordhaus and Sztorc 2016).

MARIA has been expanded to include energy technologies, land use changes with food demand and supply systems, and a simple carbon cycle model since the first development. The features of the current MARIA model are summarized as follows:

1. *Economic activity* Each region has one aggregated macro constant-elasticity-of-substitution (CES) type production function that consists of capital *K*, labor *L*, electric energy *E* and non-electric energy *N*. The putty–clay formulation is also employed. The economic output is distributed between investments *I*, consumption *C*, energy-related cost EC, trade X and the loss of economic output due to global warming DY, according to GLOBAL 2100 and DICE-2013R. The loss DY is represented by quadratic function of temperature rise similar to DICE (Nordhaus 1992).
2. *Energy flows* Eight primary energy sources, i.e., coal, oil, natural gas, nuclear power, biomass, solar power, wind power, hydraulic power, and geothermal energy, are included and converted such secondary energy types such as electricity, oil products, ethanol, methanol, hydrogen, and thermal direct use. These secondary energy carriers are further aggregated into electric and non-electric energy and then distributed among the final demand sectors, i.e., industry, transportation and other public and household sectors.
3. *Energy demand* The demand of industry sector for secondary energy is obtained by solving the inter-temporal optimization to maximize the discount summation of the utility function

12 $$U={\sum _h}{w_h}{U_h}({c_h})={\sum _h}{w_h}{\sum _t}{\left( {1 - {\delta _h}} \right)^t}{L_{h,t}}{\text{In}}\left( {{C_{h,t}}/{L_{h,t}}} \right)$$

where *h* denotes the region and the *w*_*h*_ refers to Negishi weights (Negishi 1972). The energy demands of other sectors are determined by simple demand functions with population and per capita income.

4. *Carbon circulation and climate changes* The Bern carbon cycle model (Joos et al. 2001) and a simple climate model block following DICE-2013R are incorporated. Equilibrium climate sensitivity is one of the key parameters of this block. Bern carbon cycle model and climate model deal with the simple feedback loop between temperature change and the carbon emission from biosphere.
5. *Nuclear fuel cycle* Three reactor types are considered explicitly, namely a light water reactor (LWR), an LWR with Pu fuel (LWR-Pu) and a fast breeding reactor (FBR). Thus, MARIA tends to generate nuclear-oriented solution unless policy constraints on nuclear power are imposed.
6. *Carbon capture and sequestration (CCS)* Three storage types are considered, namely aquifer, depleted gas wells and the deep ocean disposal. In MARIA, CCS can be implemented in energy sectors as power generation and industry sectors, if needed.
7. *Food demand and land use changes* MARIA deals with land use changes among cropland, forest, pasture and other types based on the yield growth of crops and animals assuming simple demand functions for these foods. The trade-offs between food production and energy crops are explicitly formulated explicitly.

The radiative forcing of non-carbon greenhouse gases (GHGs) is given exogenously. The relationships among factors can be observed briefly because the various sources of climate change in the model are connected with economic activity and energy demands.

It should be noted that the inclusion of carbon cycle and climate models result in slightly different total GHG emission pathways even if identical anthropogenic GHG emission scenarios are given under different parameter values for equilibrium climate sensitivity.

**Fig. 3:**
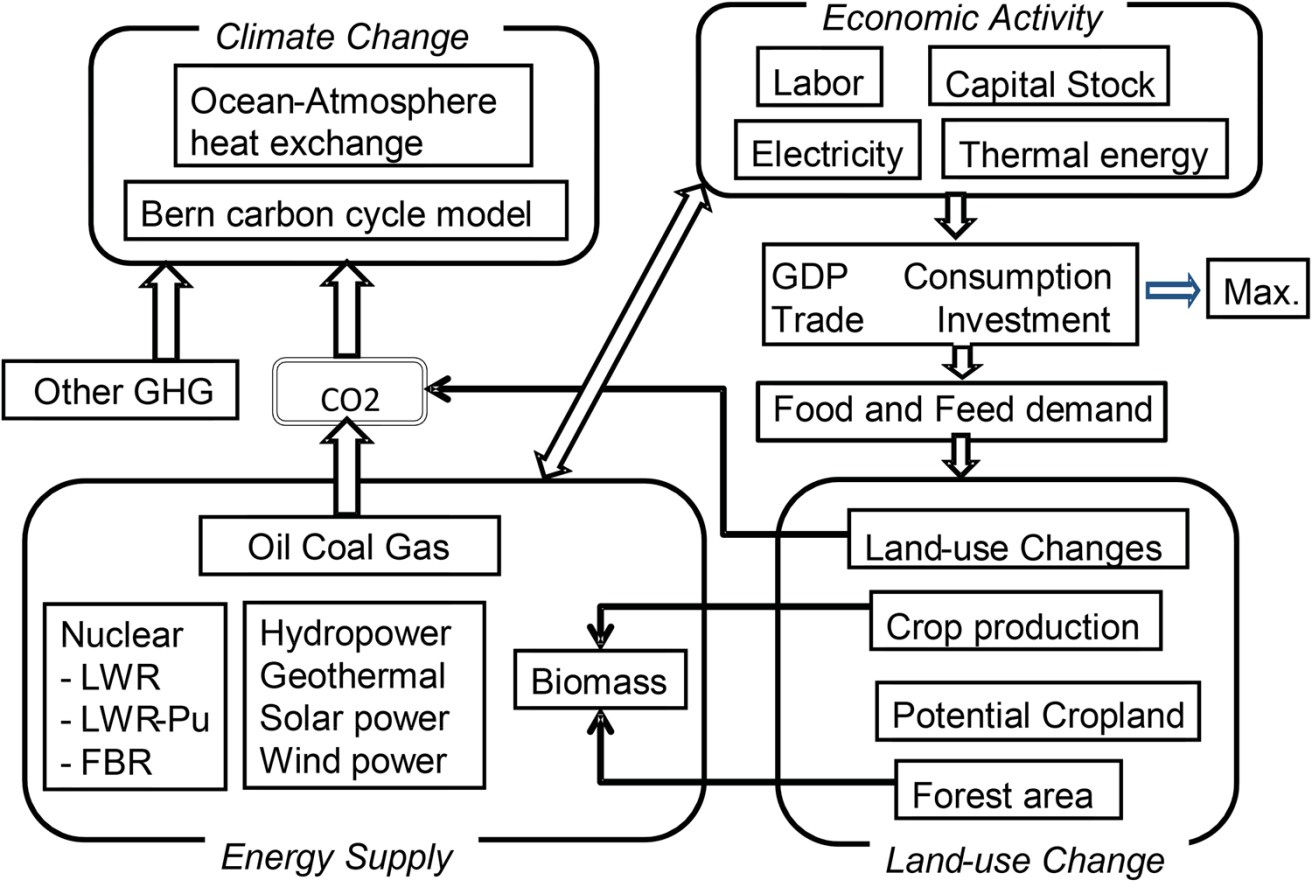
Structure of MARIA model

It is structured as shown in Fig. 3 and more details are shown in Mori et al. (2013). Herein we divide the world into five regions according to SSPs to reduce the calculation time. The full-scale MARIA specifies 23 world regions.

Before the model simulation, it is fundamental to give the key drivers such as population, economic growth rates and technological progress in production processes. Unlike the models in the natural science fields, there should be various choices and possibilities in the future society. To share the common parameters for model simulations, the world climate community has developed the shared socioeconomic pathways (SSPs) to deal with future societal possibilities including population, gross domestic product (GDP), energy, land use, and greenhouse gas emissions identifying five different future scenarios (Riahi et al. 2017; O’Neill et al. 2017). In our simulations, because AIM participated in the SSP activity and provided data set for the SSP scenarios (Fujimori et al. 2017), we use the AIM outputs for SSP2 (Middle of the road), as references. We extract AIM-output scenario data concerning population, GDP in market exchange rate, final energy consumption, and GHG emission pathways. The assumptions made about other parameters related to resource endowment, renewable energy, and CCS potentials, as well as other costs, are discussed in the existing literature (Mori 2000; Mori and Takahashi 1999; Mori et al. 2013).

### Expansion of ATL procedure

**Fig. 4:**
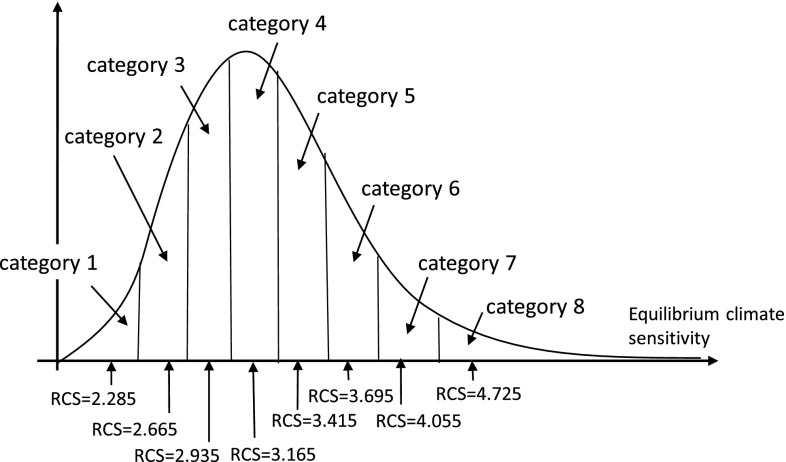
Definition of eight categories of equilibrium climate sensitivity distribution at the initial period and their representative values. *RCS denotes representative value of equilibrium climate sensitivity of the category

We begin by applying the lognormal distribution according to Lewandowski et al. (2014) to the distribution in equilibrium climate sensitivity gathered in the CMIP5 GCMs (CMIP 2017; Collins et al. 2013) to represent the uncertainty elimination process described in the previous section. This distribution represents the range of scientific knowledge regarding future climate changes at the initial period year 2010. We divide the distribution into eight sections, each of which is assumed to gives a 1/8 probability to aggregate the uncertainty. We use the median of each section of the distribution as the representative of the category. The definition of eight categories of equilibrium climate sensitivity distribution and their representative values are shown in Fig. 4. More detailed division would be preferable to better approximate the distribution. We employed eight categories because of the limit on our numerical calculation.

Following are the assumptions of the present study:

1. The “true” equilibrium climate sensitivity is within one of the eight representative values in Fig. 4;
2. In 2010, the scientific knowledge regarding equilibrium climate sensitivity dictates that the eight values in Fig. 4 are evenly possible.
3. By the continuously accumulating scientific knowledge, the uncertainty will be reduced to 3/8 by 2040 and to 2/8 by 2050. In other words, the possible range of the equilibrium climate uncertainty is narrowed from range of all eight categories to that of three categories.
4. The remaining uncertainties will persist after 2100 but eventually the “true” equilibrium climate sensitivity will be revealed.

In the initial period, the range of our scientific knowledge is represented by the set of all categories {1, 2, 3, 4, 5, 6, 7, 8}. Hereafter, we call the set of possible categories surrounded by braces as a “state”. State represents the possible range of equilibrium climate sensitivity at the certain period. The uncertainty resolving procedure in the above can be represented by the elimination of categories with probability 0. For example, the procedure {1, 2, 3, 4, 5, 6, 7, 8}–{1, 2, 3} (in 2030)–{1,2} (in 2040)–{1} (beyond 2100) constitutes one scenario. These are summarized in Table 1 where 24 scenarios are identified corresponding to the set of scenarios *S* = {s} in 2.2. The scenario probability is given as evenly as to give the final state probability 1/8 equally. For instance, only the scenario pathway {1,2,3}–{1,2}–(1} reaches final state {1}, whereas three pathways {1,2,3}–(1,2}–(2}, {1,2,3}–{2,3}–{2) and {2,3,4}–(2,3}–{2} reach finally state {2}. Similarly, four pathways reach finally state {3}. Because the probability of all final states is 1/8 equally, we allocate scenario pathway probabilities proportional to the inverse of the number of pathways which reach the certain final state.

**Table 1:** Definition of future scenarios as the state transition pathways

Scenario	Probability	State in 2040	State in 2050	Final state beyond 2100
1	1/8	{1,2,3}	{1,2}	{1}
2	1/24	{1,2,3}	{1,2}	{2}
3	1/24	{1,2,3}	{2,3}	{2}
4	1/32	{1,2,3}	{2,3}	{3}
5	1/24	{2,3,4}	{2,3}	{2}
6	1/32	{2,3,4}	{2,3}	{3}
7	1/32	{2,3,4}	{3,4}	{3}
8	1/32	{2,3,4}	{3,4}	{4}
9	1/32	{3,4,5}	{3,4}	{3}
10	1/32	{3,4,5}	{3,4}	{4}
11	1/32	{3,4,5}	{4,5}	{4}
12	1/32	{3,4,5}	{4,5}	{5}
13	1/32	{4,5,6}	{4,5}	{4}
14	1/32	{4,5,6}	{4,5}	{5}
15	1/32	{4,5,6}	{5,6}	{5}
16	1/32	{4,5,6}	{5,6}	{6}
17	1/32	{5,6,7}	{5,6}	{5}
18	1/32	{5,6,7}	{5,6}	{6}
19	1/32	{5,6,7}	{6,7}	{6}
20	1/24	{5,6,7}	{6,7}	{7}
21	1/32	{6,7,8}	{6,7}	{6}
22	1/24	{6,7,8}	{6,7}	{7}
23	1/24	{6,7,8}	{7,8}	{7}
24	1/32	{6,7,8}	{7,8}	{8}

Scenario probability is allocated as evenly as to give final state probability 1/8 equally

Note that in contrast to previous ATL studies, the intermediate uncertainty sections overlap in Table 1. In this case, the {1,2,3}–{2,3}–{2} and {2,3,4}–{2,3}-{2} paths can give different results. We must simultaneously consider 24 paths with path-dependent cases to formulate the transition flow shown in Table 1. We divide the ATL calculation into six sub-models, each containing four mutually exclusive uncertain sections to avoid exceeding the capacity of the optimization software. The six sub-models A–F and their ATL calculations are given in Table 2. Although each sub-model generates eight ATL pathways, only four (those colored light-green in Table 2) constitute part of the original ATL model. Thus, this procedure approximates the solution from 24 pathways extracted from 48 available pathways.

**Table 2:** Decomposition of calculation with six sub-models and the 24 paths to be included in the ATL analysis

	Final state	{1}	{2}	{3}	{4}	{5}	{6}	{7}	{8}
	Equilibrium climate sensitivity	2.285	2.665	2.935	3.165	3.415	3.695	4.055	4.725
Submodel-A	Stage 1	*{1,2,3}*	*{1,2,3}*	{1,2,3}	*{4,5,6}*	*{4,5,6}*	{4,5,6}	{7,8}	{7,8}
	Stage 2	*{1,2}*	*{1,2}*	{3}	*{4,5}*	*{4,5}*	{6}	{7,8}	{7,8}
Submodel-B	Stage 1	{1,2,3}	*{1,2,3}*	*{1,2,3}*	{4,5,6}	*{4,5,6}*	*{4,5,6}*	{7,8}	{7,8}
	Stage 2	{1}	*{2,3}*	*{23}*	{4}	*{5,6}*	*{5,6}*	{7,8}	{7,8}
Submodel-C	Stage 1	{1}	*{2,3,4}*	*{2,3,4}*	{2,3,4}	*{5,6,7}*	*{5,6,7}*	{5,6,7}	{8}
	Stage 2	{1}	*{2,3}*	*{2,3}*	{4}	*{5,6}*	*{5,6}*	{7}	{8}
Submodel-D	Stage 1	{1}	{2,3,4}	*{2,3,4}*	*{2,3,4}*	{5,6,7}	*{5,6,7}*	*{5,6,7}*	{8}
	Stage 2	{1}	{2}	*{3,4}*	*{3,4}*	{5}	*{6,7}*	*{6,7}*	{8}
Submodel-E	Stage 1	{1,2}	{1,2}	*{3,4,5}*	*{3,4,5}*	{3,4,5}	*{6,7,8}*	*{6,7,8}*	{8}
	Stage 2	{1,2}	{1,2}	*{3,4}*	*{3,4}*	{5}	*{6,7}*	*{6,7}*	{8}
Submodel-F	Stage 1	{1,2}	{1,2}	{3,4,5}	*{3,4,5}*	*{3,4,5}*	{6,7,8}	*{6,7,8}*	*{6,7,8}*

States are represented by the categories in the braces corresponding to Table 1

To solve the ATL procedure, we divide the model into six sub-models (sub-model A–F). Each sub-model generates solution under the specific ATL conditions shown in Eq. (3). Four of eight scenarios generated by each sub-model values given in italics constitute the part of original ATL model

We still need approximately 3 months to obtain the approximated solution of these decomposed calculations by six PCs with 3.6 GHz core i7-4770 on GAMS24.4-CONOPT. This decomposition could have reduced the calculation but the ATL constraints given by Eq. (3) is guaranteed only within each sub-model. Nonetheless, we believe this approximation still makes sense to see the extended ATL decision.

Before the calculation of ATL and single-stage decision-making processes, the policy control variables to be constrained by Eq. (3) should be defined. In this study, first we imposed the ATL constraints on the investments in the economic activities and energy technologies such as power generation expansions and the fuel demands in term of fossil fuels and biomass. Note that land use change is excluded from the ATL constraints, because it can be adapted within a decade according to the climate changes. Second, even if investment is identical, outputs can vary depending on climate changes owing to the inclusion of warming damage terms; thus, even if energy-based emissions are similarly constrained, total carbon emissions will change depending on the climate sensitivity assumption.

## Results

In the MARIA calculation with ATL, the global average temperature rise was constrained to below 2 °C from pre-industrial level. However, in the two highest equilibrium climate sensitivity cases, MARIA failed to provide feasible solutions. Thus, we relaxed the warming limitation and constrained the fuel-based carbon emission limitation in the scenarios with final state {7} and {8} to give below 2.5 °C rather than 2 °C.

**Fig. 5:**
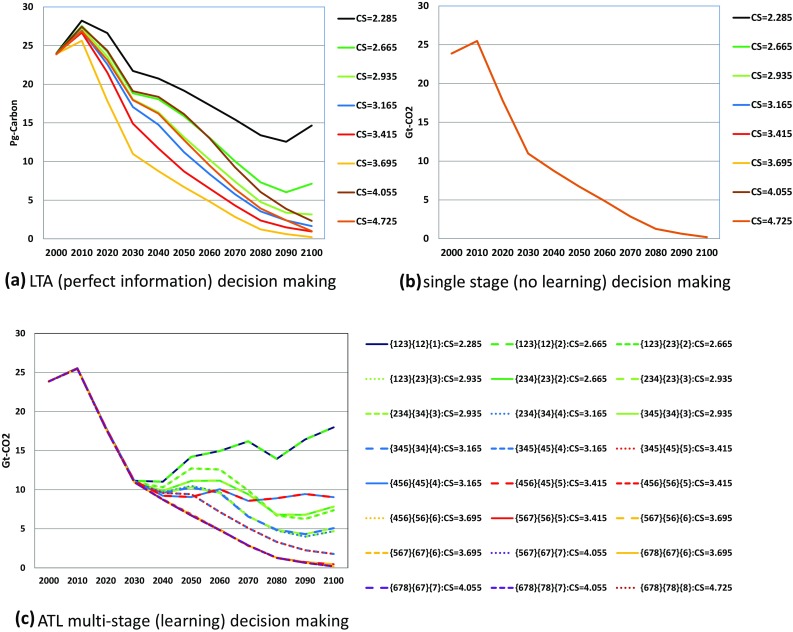
Energy-based carbon emission pathways in billion ton of CO_2_ under LTA (perfect information), single-stage (no learning) and ATL multi-stage (learning) decision-making. *CS in the figures represents true equilibrium climate sensitivity. **The line color corresponds to the equilibrium climate sensitivity value. ***In (c) ATL multi-stage (learning) decision-making, the line type represents the transition pattern of the states: {a,b,c}–{a,b}–{a} (solid line); {a,b,c}–{a,b}–{b} (broken line); {a,b,c}–{b,c}–{c} (dotted line; {a,b,c}–{b,c}–{c} (fine dotted line)

**Fig. 6:**
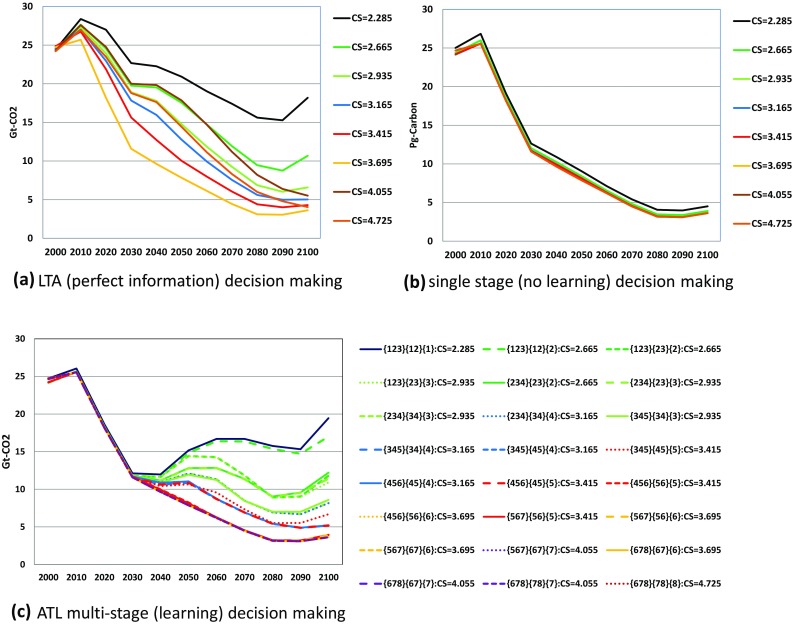
Carbon emission pathways in billion ton of CO_2_ under LTA (perfect information), single-stage (no learning) and ATL multi-stage (learning) decision-making: emission and absorption of biosphere and land use changes are included. *Line colors and line types are the same as those in Fig. 5

Comparing carbon emissions between Figs. 5 and 6 gives interesting findings. First, although single-stage energy-based emissions are identical among the cases, those with land use changes and a biosphere shown in Fig. 6b yield small differences. This is because emissions from land use changes and biosphere can vary according to the different global warming caused by the different climate sensitivities. Future climate changes and their impacts as warming damage affect the present behavior, even if energy technology implementation and economic activities such as investment are constrained to be identical among the scenarios because MARIA is an inter-temporal perfectly foreseeable optimization.

**Fig. 7:**
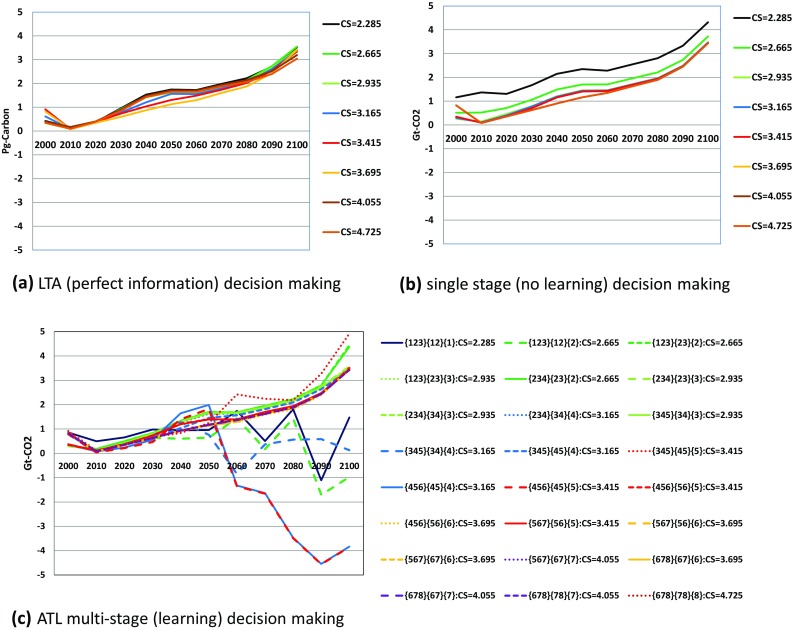
Carbon emission pathways in billion ton of CO_2_ from biosphere and land use changes under LTA (perfect information), single-stage (no learning) and ATL multi-stage (learning) decision-making. *Line colors and line types are the same as those in Fig. 5

Second, energy-based carbon emissions under LTA and single-stage decision-making in Fig. 5a, b show around 4Gt-CO_2_ higher values than those in Fig. 6a, b, respectively. As shown in Fig. 7, the carbon emissions from land use changes and a biosphere are always positive in these two cases. By contrast, in the LTA decision case, as shown in Fig. 7c, the land use changes such as afforestation work as absorption sources. This case obviously appears in {4,5,6}–{4,5}–{4} and {4,5,6}–{4,5}–{5} scenarios. This might be because final state {6} requires most stringent emission reduction because the target temperature rise in the two high-equilibrium climate sensitivity categories {7} and {8} is relaxed to 2.5 °C. Therefore, the land use change policy should have been most serious in {4,5,6} case in which 2.0 °C target is needed against high climate sensitivity. When the climate change appears not so serious in the second stage, the policy could work as carbon sink.

**Fig. 8:**
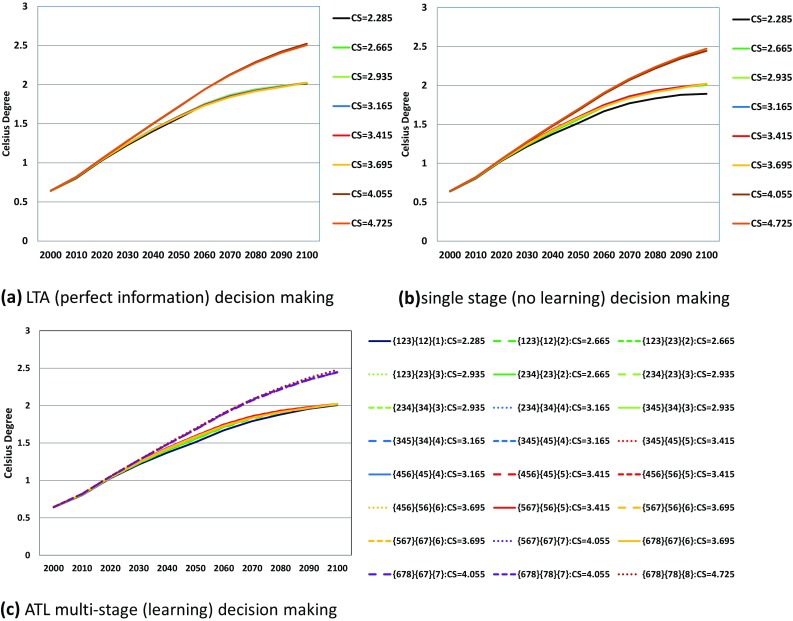
Atmospheric temperature pathways in Celsius degree under LTA (perfect information), single-stage (no learning) and ATL multi-stage (learning) decision-making. *Line colors and line types are the same as those in Fig. 5

Figures 5 and 6 compare the optimal carbon emission pathways in LTA (perfect ex ante information), single-stage decision-making (no learning), and the ATL process. In the low-equilibrium climate sensitivity scenarios, the carbon emission results largely depend on the decision-making process. Note that single-stage decision-making yields the same emission pathways from energy in all equilibrium climate sensitivity scenarios, despite the different emissions from land use among the equilibrium climate sensitivity cases. This explains the small differences in Fig. 6 for the single-stage decision case. Figure 8 compares the atmospheric temperature changes in LTA, ATL, and single-decision-making. In the highest equilibrium climate sensitivity scenarios (Scenarios 7 and 8), the temperature rise from pre-industrial levels in 2100 is approximately 2.5 °C. Conversely, the atmospheric temperature rise in the low-equilibrium climate sensitivity cases predicted by single-stage decision-making is approximately 1.85 °C. Figures 5 and 6 suggest that single-stage decision-making tends to recommend lower emission pathways than do the other prediction processes.

**Fig. 9:**
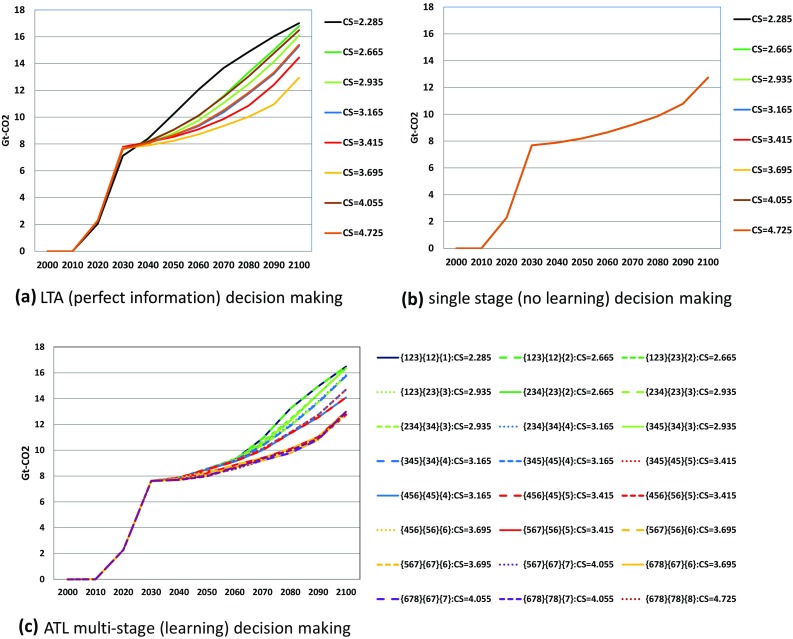
CCS implementation strategies in billion ton of CO_2_ under LTA (perfect information), single-stage (no learning) and ATL multi-stage (learning) decision-making. *Line colors and line types are the same as those in Fig. 5

We focus on the CCS implementation patterns and biomass energy demands among the cases to determine how the technology strategy changes under the different decision-making procedures. The role of CCS in the Paris Agreement has been pointed out in many literature sources including IPCC-AR5-WG3 (2014a), this being despite the high barrier to large scale deployment (IPCC-AR5-WG3 2014b). The expansion of CCS should be planned carefully because little co-benefit of CCS implementation is expected. Figure 9 shows the CCS implementation results in the different decision-making cases. First, decision-making under uncertainty preferred minimum implementation of CCS because CCS is expensive and lowers the energy conversion efficiency. Second, the temperature rise at the end of the twenty-first century can exceed 2 °C in the two highest climate sensitivity cases {7} and {8}. There is less need for CCS in these cases, and so CCS in single-stage decision-making is implemented moderately.

**Fig. 10:**
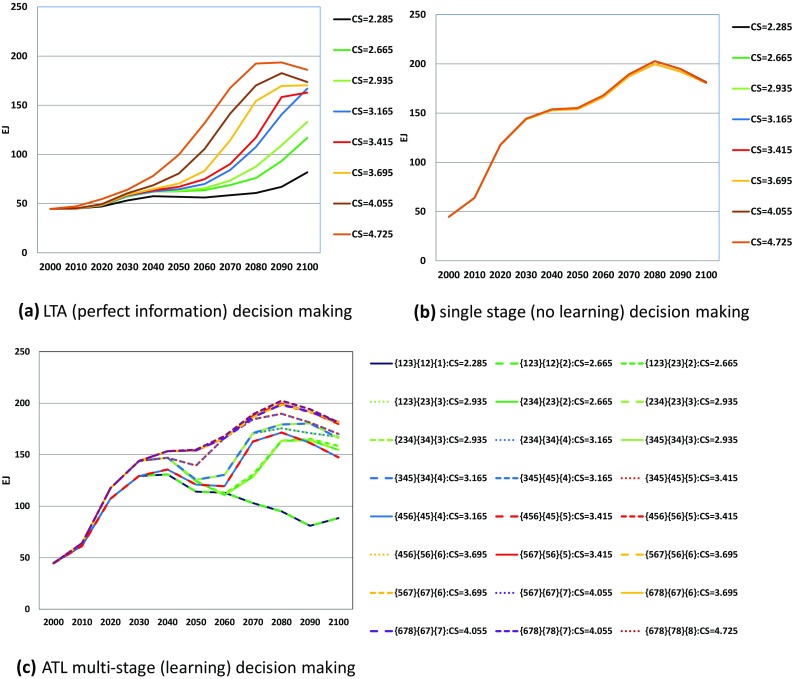
Biomass primary energy supply pathways in EJ under LTA (perfect information), single-stage (no learning) and ATL multi-stage (learning) decision-making. *Line colors and line types are the same as those in Fig. 5

Figure 10 compares the biomass energy utilization in the different decision-making cases. In contrast to the case of CCS, biomass demand in the single-stage decision-making (Fig. 10b) is higher than that in the LTA perfect-information case (Fig. 10a). So long as high future global warming is possible, the suggestion is to prioritize the expansion of biomass. The results for ATL yield fluctuating patterns. As suggested in Fig. 10a, using biomass as an energy source is not the first priority when climate change is low. If multi-stage decision-making is available, biomass utilization should be implemented flexibly according to the knowledge acquisition.

**Fig. 11:**
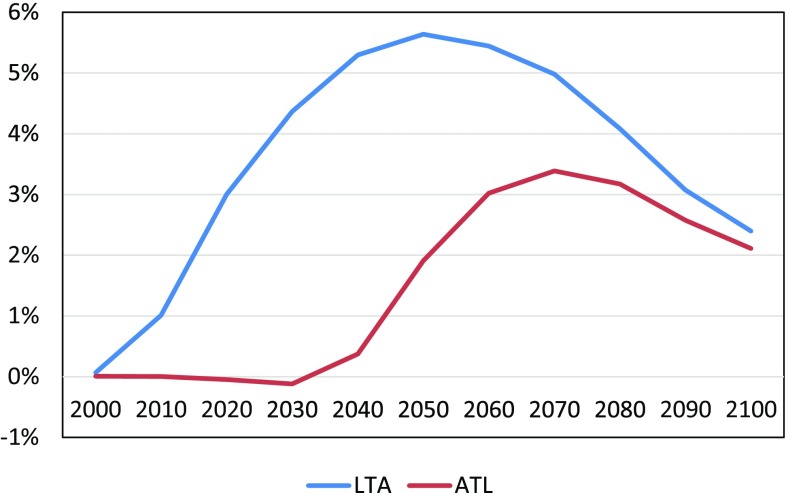
Ratio of information value VPIR(*t*) under LTA (perfect information) and VLP(*t*) under ATL multi-stage (learning) decision-making to the GDP of single-stage decision-making

Figure 11 compares the trajectories of the value of information in LTA and ATL, i.e., VPIR(*t*) and VLPR(*t*) in Eqs. (6) and (10), respectively. The value of perfect information already appears in the beginning of the twenty-first century whereas those in learning case become apparent in the second half of this century according to the assumption in the uncertainty elimination process. However, the contribution of knowledge accumulation still exceeds 3%.

Table 3 summarizes the changes in cumulative GDP with different decision-making processes and different targets. The information values and their contribution ratios, TVPI, TVPIR, TVLP and TVLPR are calculated based on the changes in cumulative GDP between the learning and no-learning (single-stage decision-making) cases. We calculated TVPI and TVLP under zero discount rate. It is well known that the assumption on the discount rate is highly controversial (IPCC-AR4-WG3 2007). The discounted present value is often calculated under a discount rate of 5%, whereas discount rate of 1.5% is widely used for the discounting the social time preference utility in the integrated assessment models (Nordhaus 2014; Mori et al. 2013). However, because the outcomes of scientific research often appear after many years later, unlike the investment and return of certain business project, it is questionable whether the concept of “discounted present value” is directly applicable. Thus, we tentatively adopted a zero discount rate when evaluating the average contribution of scientific information.

**Table 3:** Changes in cumulative GDP in 2005 USD during the 2000–2100

Target	Decision-making	Cumulative GDP in 2005 trillion $	Value of information in 2005 trillion $	Per year value of information in 2005 billion $	Value of information in % of GDP
2.5 °C	LTA:Full information	14589.2	116.08	1289.8	0.802%
	ATL:Learning information	14581.7	108.53	1205.9	0.750%
	Single Stage	14473.2	–	–	–
2.0 °C	LTA:Full information	14163.5	528.43	5871.5	3.876%
	ATL:Learning information	13933.2	298.12	3312.4	2.186%
	Single stage	13635.1	–	–	–

From Table 3, we find that the value of information increases as the climate target policy becomes more stringent, being approximately 4.5 times higher with a 2.0 °C target than with a 2.5 °C target. Furthermore, the value of learning information with a 2.0 °C target is just over 3.31 trillion 2005 US dollars per year in ATL, while 5.87 trillion 2005 US dollars per year when full information is available from the beginning. Thus, scientific knowledge is valuable even when the uncertainties in equilibrium climate sensitivity are not fully resolved. In other words, expenditure on scientific research is rationalized when global climate changes are of true concern.

Table 4 summarizes the CCS implementation results of the 24 pathways analyzed in the ATL multi-stage decision-making. This table shows the path-dependency effects described in 3.2.

**Table 4:**
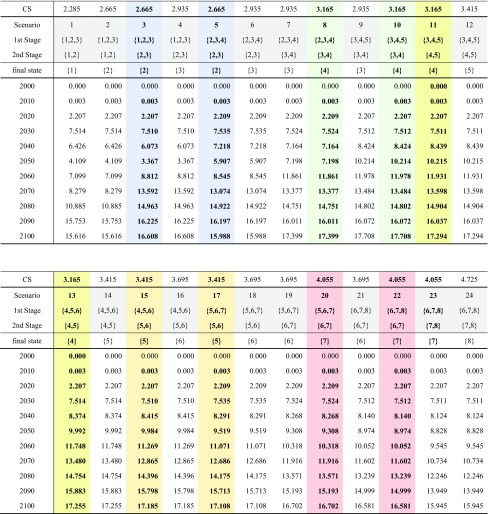
Results of CCS implementation in billion tons of CO_2_ in ALT multi-stage decision case

The columns filled by the same color indicate the same state in stage 2 with identical true climate sensitivity, whereas the states in stage 1 are different

The second, the third and the fourth rows indicate the states in 2040, 2050 and the true climate sensitivity revealed beyond 2100 corresponding to Table 1, respectively

## Discussion

The purpose of this study was to address the optimal emission pathways toward the Paris Agreement before we have perfect information about climate changes, and to value the acquisition of scientific information. We expanded the existing multi-stage decision-making process and applied it to the MARIA integrated assessment model, employing the findings provided by the ASK method.

For the first purpose, the simulation results as shown in Fig. 5b reveal the need to minimize the CO_2_ emissions in pathways unless future information on climate change is provided. By comparing Fig. 5a, c, we see that the CO_2_ emission pathways should be constrained around 12 Gt-CO_2_ by 2030 showing that the CO_2_ emission in 2000 should be cut almost 50% in 2030. This is because of the possibility of global warming that is higher than the median or mean climate sensitivity case. In other words, the decision of the climate policy should not be delayed if we are serious about adopting a stringent global-warming target. Relaxation of emission constraints is possible only if new scientific knowledge is acquired. If we can exclude the high climate sensitivity case, the constraint could also be relaxed before 2030.

As Fig. 6 suggests the total greenhouse gas emission fluctuates slightly because of the emissions from non-energy sources. The uncertainties in the emissions from non-energy sources and the mitigation options for these sources are limited. This issue becomes serious when we consider a 1.5 °C target because zero carbon emission would be insufficient. Thus, uncertainties in the emissions and mitigation options of non-energy sources should be evaluated in the next stage of this research.

Figures 9 and 10 show the opposite properties of mitigation options. In single-stage decision-making, CCS is introduced at the minimum value given by LTA decision-making whereas biomass is adopted at the maximum value. CCS is implemented considering the lowest warming case, whereas biomass is not adopted. It should be noted that biomass utilization fluctuates complicatedly according to knowledge acquisition, as shown in Fig. 10c. Such fluctuations might imply short-term land-use changes because the expansion of biomass is strongly related to land-use changes as well as to food demand. Even if MARIA were to include a simple calculation of the emissions due to land-use changes, more detailed investigation would be needed to assess the possibility of biomass expansion.

**Fig. 12:**
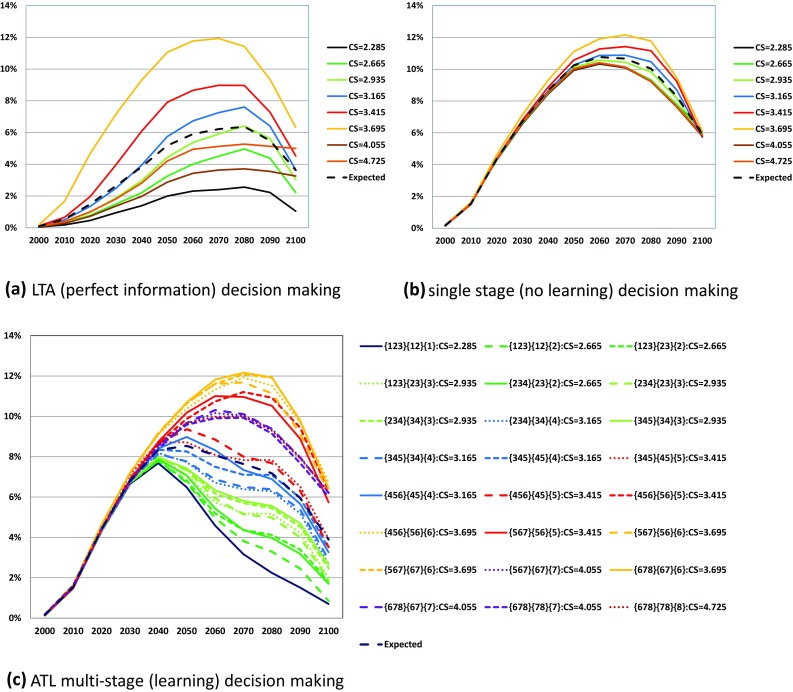
GDP loss from BAU pathways under LTA (perfect information), single-stage (no learning) and ATL multi-stage (learning) decision-making. *Line colors and line types are the same as those in Fig. 5

Valuing information should be considered further because we have few existing studies (Manne and Richels 1992). We cannot directly compare our evaluation with existing studies. However, there are many studies that evaluate the GDP loss or costs of climate policies by comparing the differences in GDP with and without a climate policy or focus on the carbon prices. For the former approach, IPCC-AR5-WG3 (IPCC-AR5-WG3 2014c) reports the loss of GDP under various carbon-control scenarios, showing a GDP loss of 1.8–15% (around 5% median) in 2100 in the case of a carbon-equivalent 450-ppmv concentration constraint. We show the trajectories of the rates of GDP loss for our three decision-making cases in Fig. 12.

Figure 12 shows that the maximum GDP losses range from 2 to 12% corresponding to the equilibrium sensitivity values, while the GDP loss in terms of the expected value of LTA in Fig. 12a is approximately 6%. When we have no learning, as shown in Fig. 12b, the GDP loss is 10–12%. The maximum GDP loss appears at 3.695 °C equilibrium climate sensitivity in all cases whereas the GDP loss declines as the information is acquired. When we compare the maximum expected GDP loss among the cases, LTA decision-making, single-stage without learning, and ATL learning give 6, 11, and 8.5%, respectively. Because we do not know the “true” equilibrium sensitivity today, it could be said that the GDP in Fig. 12b is the current appropriate evaluation of GDP loss to maintain a 2 °C target today and that the differences between Fig. 12b and c will be eliminated as scientific knowledge is accumulated. It is not easy to compare these numbers to those in the real world. For example, Whitehouse (2013) reports an expenditure of $21.4 billion (current USD) on climate change research, which is 0.1% of GDP. Such a comparison may be meaningless. However, the value of scientific knowledge should not be underestimated.

## Conclusions

This study evaluated the contribution of accumulated scientific knowledge to the economic loss of mitigating climate change. The novel ASK method of Shiogama et al. (2016) and its findings on the uncertainty elimination procedure were applied to ATL decision-making.

We assessed how the CO_2_ emission pathways are affected by different decision-making procedures. The simulation shows that the emissions should be minimized without information. The implementation of CCS and biomass shows different patterns, whereby CCS is implemented at its minimum level without learning. CCS implementation increases as the information on the possibility of higher climate sensitivity reveals. In contrast, biomass is expanded at its maximum level and then decreases as the possibility of lower climate sensitivity appears.

We estimated that the value of full information with a 2.0 °C target is approximately 4.5 times of that with a 2.5 °C. With a 2.0 °C target case, the value of learning information, which is defined as the mitigation of GDP, is almost 3.31 2005 US trillion dollars (approximately 2.19% of GDP without learning) per year while that with full information from the beginning is 5.87 2005 US trillion dollars (approximately 3.88% of GDP without learning). Comparing these values with the losses of GDP under the 450-ppmv control scenario from GDP without climate policy reported in IPCC-AR5-WG3 (IPCC-AR5-WG3 2014c), the value of information indicates almost half of GDP losses between BAU and climate policy case. Thus, we can conclude that the scientific knowledge is still valuable even if the uncertainties in the climate sensitivity are not fully resolved.

Since the Paris Agreement has taken effect, the effects and possibility of a 1.5 °C target have been widely discussed as a more preferable target, although its difficulty under current technology has also been pointed out. In fact, the multi-model approach adopted by the ICA-RUS project shows that two of four integrated assessment models (including MARIA) could not find feasible solutions under the 1.5 °C target with a 3.6 °C equilibrium climate sensitivity for the SSP2 scenario (Mori et al. 2017). In this study, MARIA could not find feasible solutions for the 1.5 °C target when the equilibrium climate sensitivity was larger than 3.0 °C. We should consider additional innovative technological options even if large uncertainty is expected to accommodate the possibility of a 1.5 °C target.

Note that the evaluation by IAMs covers only market economic losses. Because the uncertainties in climate change may be much larger than expected, the societal and ecological losses could significantly exceed the economic loss. From a risk-management viewpoint, the uncertainty evaluation warrants further investigation.
